# The Moderating Role of Psychological Well‐Being in the Relation Between Stressful Life Events and Common Mental Disorders in the General Population

**DOI:** 10.1111/sjop.70061

**Published:** 2025-12-03

**Authors:** Peter M. ten Klooster, Margreet ten Have, Annemarie I. Luik, Marlous Tuithof, Ernst T. Bohlmeijer

**Affiliations:** ^1^ Department of Technology, Human and Institutional Behaviour University of Twente Enschede the Netherlands; ^2^ Netherlands Institute of Mental Health and Addiction (Trimbos Institute) Utrecht the Netherlands; ^3^ Department of Epidemiology Erasmus MC University Medical Centre Rotterdam the Netherlands

**Keywords:** mental disorders, population health, psychological well‐being, risk factors, stressful events

## Abstract

Stressful life events (SLEs) are known to be associated with an increased prevalence of common mental disorders (CMDs), but the potential moderating role of psychological well‐being has not been comprehensively studied. In total, 6194 adults aged 18–75 years were interviewed for the third Netherlands Mental Health Survey and Incidence Study (NEMESIS‐3). Assessments included the adapted Composite International Diagnostic Interview (CIDI v3.0) to determine DSM‐5 mood, anxiety and substance use disorders, Brugha's List of Threatening Experiences for SLEs, and Brief INSPIRE‐O for psychological well‐being. Logistic regressions tested associations between having experienced at least two SLEs and the different CMDs and additive interactions with psychological well‐being. Having experienced ≥ 2 SLEs in the last year was associated with a higher prevalence of all CMDs in the last year, with adjusted odds ratios ranging from 1.71 (95% CI: 1.39; 2.10) for substance use disorders to 3.43 (95% CI: 2.73; 4.30) for mood disorders. The interaction effect of ≥ 2 SLEs and low psychological well‐being was statistically significant for any CMD (RERI = 5.64, 95% CI: 3.18; 8.10), mood disorder (RERI = 23.09, 95% CI: 10.10; 36.10) and anxiety disorder (RERI = 3.45, 95% CI: 1.27; 5.63), but not for substance use disorder (RERI = 0.21, 95% CI: −1.38; 1.80). The joint presence of ≥ 2 SLEs and poor psychological well‐being was associated with a higher prevalence of mood and anxiety disorders than would be expected from the sum of their individual associations. Promoting psychological well‐being may be a fruitful public mental health strategy to increase resilience against SLEs.

**Summary**

- Experiencing at least 2 serous life events in the past year is associated with higher odds of meeting DSM‐5 criteria for anxiety, mood and substance use disorder in the same year.
- Having poor psychological well‐being carries an excess risk for mood and anxiety disorder, but not for substance use disorder, for people who experienced multiple serious life events.
- Promoting psychological well‐being in the general population may be a fruitful public mental health strategy to increase the resilience against SLEs.

## 1 Introduction

Life events have been long recognized as important risk factors for the development and course of mental illness. Community studies have shown stressful life events (SLEs) to be associated with an increased prevalence of common mental disorders (CMDs), including mood, anxiety, and substance use disorders (Clark et al. 2012; Jordanova et al. 2007; Newman and Bland 1994; Verplaetse et al. 2018). Although associations between recent SLEs and CMDs are well established at the group level, not all people who experience SLEs develop mental health problems. Previous studies have explored different moderating factors that could promote positive adjustment or resilience to SLEs. Often grounded in the stress‐vulnerability model of mental illness, studies demonstrated the protective role of perceived self‐control or coping skills (Asselmann et al. 2017) and other resilience resources, such as optimism and mastery (Julian et al. 2021) and spirituality (Young et al. 2000), against different mental disorders. Other studies have demonstrated a buffering role of perceived social support or connectedness (Yu and Liu 2021; Wagner et al. 1996).

Most of these moderators or resilience resources are closely linked to the basic domains of positive psychological functioning and well‐being. Ryff (1989) elaborated the concept of psychological well‐being as consisting of six elements of positive mental functioning: self‐acceptance, purpose in life, autonomy, positive relationships with others, environmental mastery, and personal growth. Each element is important in striving to live a meaningful and fulfilled life. Many studies have found positive associations between psychological well‐being and indicators of physical and mental health and longevity, underscoring the potential protective features of psychological well‐being (Ryff 2013). For example, higher levels of psychological well‐being have been longitudinally linked to reduced risk of major depressive disorder and incident mood, anxiety, and substance use disorders (Keyes et al. 2010; Wood and Joseph 2010; Schotanus‐Dijkstra et al. 2017).

Various instruments measuring positive mental functioning have been developed, such as Ryff's psychological well‐being scale (Ryff 1989; Ryff and Keyes 1995) and Keyes' mental health continuum‐short form (Keyes 2002). Another emerging instrument originally intended to measure positive mental functioning in the context of personal recovery from mental illness is the INSPIRE scale (Williams et al. 2015). Personal recovery has been defined as “a way of living a satisfying, hopeful and contributing life even with the limitations caused by illness” (Anthony 1993). Leamy et al. (2011) developed the CHIME framework of personal recovery, comprising five domains: connectedness, hope and optimism about the future, identity, meaning in life, and empowerment. The INSPIRE scale was developed to measure these five CHIME domains, and both the original version and a 5‐item short‐form version (Brief INSPIRE) demonstrated good psychometric properties (Williams et al. 2015). Moeller et al. (2023, 2024) recently developed the Brief INSPIRE‐O version, which can be used outside mental healthcare settings. Conceptually, the CHIME domains strongly overlap with the components of psychological well‐being (Ruan et al. 2025), and it has been argued that recovery is not only relevant for people with mental health issues, but constitutes a unifying human experience (Slade 2010; Van Eck et al. 2023). Personal recovery scores have previously indeed been shown to be strongly correlated with psychological well‐being (Kraiss et al. 2019) and similarly associated with factors such as positive self, positive action, and social functioning in people with non‐affective psychosis, their siblings, and a healthy control group (Van Eck et al. 2023).

Although the individual links of both SLEs and psychological well‐being with CMDs are well established, no studies have specifically examined the interaction between SLEs and psychological well‐being as an integrated, overarching construct. Instead, available studies have focused only on the potential moderating role of specific resources that are conceptually similar to specific facets of psychological well‐being, such as positive connections, optimism, self‐acceptance, meaning in life, and perceived self‐control and mastery (Asselmann et al. 2017; Julian et al. 2021; Yu and Liu 2021; Wagner et al. 1996; Dulaney et al. 2018; Chou and Chi 2001). Moreover, these studies primarily relied on self‐reported symptoms as the dependent variable, rather than on clinically diagnosed disorders assessed with a validated diagnostic interview. Finally, most studies examined these interaction effects only in specific subgroups of the population, such as adolescents or the elderly. If the combined occurrence of SLEs and low psychological well‐being is consistently observed to coincide with a higher prevalence of CMDs in the general population, this would highlight the potential relevance of psychological well‐being as a target for public health strategies aimed at strengthening resilience in the face of SLEs. In the current cross‐sectional study, we first examined the association between recently experienced negative life events and the presence of CMDs in the past 12 months in a large and representative sample of the adult Dutch population aged 18–75 years. Second, the moderating role of psychological well‐being, as measured by the Brief INSPIRE‐O, in the association between negative life events and the presence of CMDs was explored.

## 2 Methods

### 2.1 Study Design

Data were drawn from the first wave of the third Netherlands Mental Health Survey and Incidence Study (NEMESIS‐3) (ten Have et al. 2023). NEMESIS‐3 is a cohort sampled from the Dutch general population, mainly focused on providing up‐to‐date information on the prevalence, incidence, course, risk factors, and consequences of mental disorders. Participants were selected using a multistage sampling procedure. First, a stratified random sample of 148 of the 355 Dutch municipalities (with the four largest cities pre‐included) was drawn. Next, a random sample of individuals aged 18–75 years was drawn from these municipalities using the Personal Records Database (BRP). Individuals with insufficient command of the Dutch language or who were institutionalized were excluded. Individuals who returned home from being institutionalized during the fieldwork could, however, be interviewed later. For the first wave of NEMESIS‐3, the recruitment resulted in 6194 adults who were interviewed face‐to‐face at their homes between November 2019 and March 2022. Compared to the sociodemographic characteristics of the total Dutch population, the sample was reasonably nationally representative, although some groups (e.g., people of non‐Dutch origin) were somewhat underrepresented (ten Have et al. 2023).

### 2.2 Measures

#### 2.2.1 Diagnostic Assessment

The presence of DSM‐5 CMDs in the past 12 months was measured with a slightly adapted version of the Composite International Diagnostic Interview (CIDI) version 3.0 as used in NEMESIS‐2 (de Graaf et al. 2010). The CIDI 3.0 is a structured lay‐administered diagnostic interview developed for use in the WHO World Mental Health Survey Initiative (Kessler and Üstün 2004). Adaptations were needed to determine DSM‐5 disorders, since the CIDI 3.0 was originally developed to assess DSM‐IV disorders. Details on the exact adaptations made in the CIDI 3.0 can be found elsewhere (ten Have et al. 2023). Studies of earlier CIDI versions showed that the CIDI 3.0 assesses common mental disorders with generally good validity compared with blinded clinical reappraisal interviews with the SCID for DSM‐IV (Haro et al. 2006). In NEMESIS‐3, the following CMDs were assessed: mood disorders (major depressive disorder, persistent depressive disorder, bipolar disorder), anxiety disorders (panic disorder, agoraphobia, social anxiety disorder [social phobia], specific phobia, generalized anxiety disorder), and substance use disorders (alcohol and drug use disorders).

#### 2.2.2 Stressful Life Events

The occurrence of 10 recent stressful life events (SLEs) was assessed based on Brugha's life event section (Brugha et al. 1985). Brugha's brief list of categories of SLEs has been shown to account for the vast majority of life events with an etiologically significant rating of marked or moderate long‐term threat (Brugha et al. 1985). Examples are the death of a relative or friend, divorce, and financial difficulties. Respondents indicated whether or not they experienced each event (category) in the last 12 months.

#### 2.2.3 Psychological Well‐Being

The Brief INSPIRE measures positive psychological functioning in terms of “living a satisfying and hopeful life” and consists of one item for each of the five CHIME domains identified in a systematic review (connectedness, hope, identity, meaning, empowerment) (Williams et al. 2015). Each item is rated on a 5‐point Likert‐type response scale ranging from “not at all” (0) to “very much” (4). Item scores are summed and multiplied by 5 to obtain a total score ranging from 0 to 100, with higher scores indicating higher recovery or psychological well‐being.

The original Brief INSPIRE was developed for use with mental health service users and specifically asks to what extent people feel that each CHIME domain is supported by their care provider. To allow the measure to be used in general populations, Moeller et al. (Moeller et al. 2023, 2024) changed the wording to capture an individual's experience of personal recovery, rather than recovery support. For example, “My worker helps me to feel supported by other people” was modified to “I feel supported by other people”. This version, referred to as the Brief INSPIRE‐O, demonstrated adequate internal consistency (*α* = 0.83) in their random sample of adult Danish residents, similar to the internal consistency (*α* = 0.86) reported by Williams et al. (2015) in community mental health users. In the current study, Cronbach's α was 0.75.

#### 2.2.4 Sociodemographics

Sociodemographic variables included as covariates in the current study were sex, age, educational attainment, living situation (with or without partner), and employment situation (paid employment or not).

### 2.3 Statistical Analyses

All statistical analyses were performed with Stata version 16.1. To facilitate the generalization of the data to the Dutch general population, based on post‐stratification, a weighting factor was constructed. After weighting, the distribution of the sociodemographic characteristics of the study sample was very close to that of the Dutch population (ten Have et al. 2023).

To facilitate interpretation of the association between SLEs and CMDs and the moderating role of psychological well‐being, both the number of SLEs (0–10) and psychological well‐being (0–100) were categorized. SLEs were dichotomized into having experienced at least two SLEs (24.5%) or less (reference category: 75.5%). This cutoff was chosen because more than half of the sample experienced at least one SLE and because a previous study suggested that multiple exposures to life events provided more reliable associations with psychopathology than exposure to at least one event (Tsakanikos et al. 2007). As no relevant cutoffs have yet been established for the Brief INSPIRE‐O, observed scores were categorized into the lowest 25% (first quartile; 0–65), the middle 50% (interquartile range; 66–80), and the highest 25% (fourth quartile, 81–100).

Associations between experiencing at least two SLEs in the past 12 months and the presence of different CMDs were estimated using a series of logistic regression analyses, adjusted for sex, age, educational attainment, living situation, and employment situation. Associations were expressed as odds ratios (ORs) with 95% confidence intervals. To test whether the association between at least two SLEs and the presence of a CMD was moderated by psychological well‐being, additive interaction analyses were performed. Additive interaction exists if the combined effect of two risk factors (i.e., SLEs and low psychological well‐being) on CMD is larger than the sum of the individual effects (Knol et al. 2011). Additive interaction effects were expressed as relative excess risks due to interaction (RERIs), adjusted for the demographic covariates, with 95% confidence intervals using the nlcom command in Stata (VanderWeele and Knol 2014). RERI statistics of zero indicate no interaction, or exact additivity, while positive RERI statistics imply super‐additive interaction, or excess risk.

Because both the prevalence of CMDs and the exposure to and impact of specific SLEs can vary substantially across life stages (Asselmann et al. 2019; Cohen et al. 2019; Jorm 2000; Scott et al. 2008), all association and interaction analyses were repeated stratified by age group. For this, the sample was divided into participants aged < 50 years and those ≥ 50 years, as this cutoff is commonly used to distinguish younger from middle‐aged and older adults and also corresponded closely to the sample's median age. Finally, to explore the suitability of the distribution‐based categories of psychological well‐being and to gain more detailed insight into if and how interaction effects may vary across the entire range of well‐being, additional sensitivity interaction analyses were performed in the total sample with continuous Brief INSPIRE‐O scores as the moderator.

## 3 Results

### 3.1 Sample Characteristics

Table 1 shows the descriptive characteristics of the total sample and stratified by age and sex. The mean age of the total sample was 46.2 (SD = 16.3) years. A quarter of the respondents had a CMD in the last 12 months, with anxiety disorders being the most prevalent. In total, 6.1% of the sample was prescribed psychotropic medication (mainly antidepressants or benzodiazepines) in the previous 12 months by a mental health professional. Twenty‐five percent of the sample experienced at least two SLEs in the last year. CMDs were more prevalent among participants aged < 50 years and, with the exception of substance use disorder, among women. SLE exposure rates were relatively similar across age and sex groups. Mean Brief INSPIRE‐O scores in the total sample were reasonably normally distributed and centered around more positive scores, with a mean score of 74.4 (SD = 14.4) in the total sample.

**TABLE 1:** Descriptive characteristics (unweighted n and weighted percentages) of the NEMESIS‐3 sample.

	Total sample	Aged < 50 years	Aged ≥ 50 years	Men	Women
Sex, *n* (%)					
Men	3071 (49.96%)	1504 (50.45%)	1567 (49.37%)	NA	NA
Women	3123 (50.04%)	1581 (49.55%)	1542 (50.63%)	NA	NA
Educational attainment, *n* (%)					
Primary education, lower secondary	1367 (23.16%)	372 (13.87%)	995 (34.14%)	683 (23.73%)	684 (22.60%)
Higher secondary	2259 (42.21%)	1192 (45.04%)	1067 (38.87%)	1122 (41.71%)	1137 (42.72%)
Higher vocational, university	2568 (34.62%)	1521 (41.09%)	1047 (26.99%)	1266 (34.57%)	1302 (34.68%)
Living situation, *n* (%)					
With partner	4163 (62.98%)	1820 (55.30%)	2343 (72.06%)	2059 (62.17%)	2104 (63.79%)
Without partner	2031 (37.02%)	1265 (44.70%)	766 (27.94%)	1012 (37.83%)	1019 (36.21%)
Employment situation, *n* (%)					
Paid job	4181 (69.01%)	2532 (80.86%)	1649 (55.01%)	2169 (72.80%)	2012 (65.23%)
No paid job	2013 (30.99%)	553 (19.14%)	1460 (44.99%)	902 (27.20%)	1111 (34.77%)
CMD prevalence in past year, *n* (%)					
Any disorder	1507 (25.90%)	964 (32.29%)	543 (18.35%)	676 (23.99%)	831 (27.80%)
Mood disorder	550 (9.82%)	350 (12.27%)	200 (6.93%)	216 (8.09%)	334 (11.54%)
Anxiety disorder	878 (15.24%)	536 (18.23%)	342 (11.72%)	307 (11.04%)	571 (19.45%)
Substance use disorder	418 (7.15%)	318 (10.47%)	100 (3.23%)	272 (9.50%)	146 (4.80%)
Number of SLEs in past year, *n* (%)					
0	2916 (46.73%)	1516 (48.30%)	1400 (44.86%)	1512 (49.12%)	1404 (44.34%)
1	1762 (28.26%)	845 (27.29%)	917 (29.40%)	842 (27.25%)	920 (29.26%)
≥ 2	1516 (25.02%)	724 (24.41%)	792 (25.74%)	717 (23.63%)	799 (26.40%)

Abbreviations: CMDs, common mental disorders; SLEs, stressful life events.

### 3.2 Association of SLEs and Psychological Well‐Being With CMDs

Having experienced at least two SLEs in the last year was associated with a significantly higher prevalence of all CMDs in the last year (Table 2). Adjusted odds ratios ranged from 1.71 (95% CI: 1.39; 2.10) for substance use disorders to 3.43 (95% CI: 2.73; 4.30) for mood disorders. The odds ratio for having any disorder in the past year was 2.34 (95% CI: 2.01; 2.73). Low psychological well‐being was also significantly associated with all CMDs (Table 3), again, most strongly with mood disorder. People with low psychological well‐being had an almost fivefold higher odds (OR = 4.84, 95% CI: 3.91; 6.00) compared to people with medium psychological well‐being and 78% higher odds of substance disorder (OR = 1.78, 95% CI: 1.35; 2.33).

**TABLE 2:** Associations of stressful life events with 12‐month prevalence of common mental disorders in the total sample.

	0‐1 SLEs	≥ 2 SLEs	OR (95% CI)
	*N* (%)	*N* (%)	
Any disorder	946 (21.34%)	561 (39.57%)	2.34 (2.01; 2.73)***
Mood disorder	269 (6.38%)	281 (20.12%)	3.43 (2.73; 4.30)***
Anxiety disorder	548 (12.43%)	330 (23.70%)	2.03 (1.69; 2.43)***
Substance use disorder	276 (6.19%)	142 (10.02%)	1.71 (1.39; 2.10)***

*Note:* OR = odds ratio (adjusted for sex, age, educational attainment, living situation, and employment situation).

*** *p* < 0.001.

**TABLE 3:** Associations of psychological well‐being with 12‐month prevalence of common mental disorders in the total sample.

	Psychological well‐being			OR (95% CI)	
	High	Middle	Low	High versus middle	Low versus middle
	*N* (%)	*N* (%)	*N* (%)		
Any disorder	296 (19.0%)	597 (20.9%)	614 (42.1%)	0.71 (0.60; 0.84)***	2.92 (2.44; 3.49)***
Mood disorder	63 (4.1%)	163 (5.8%)	324 (22.9%)	0.56 (0.40; 0.80)**	4.84 (3.91; 6.00)***
Anxiety disorder	158 (10.2%)	329 (11.5%)	391 (27.2%)	0.74 (0.63; 0.88)***	2.84 (2.31; 3.48)***
Substance use disorder	94 (5.9%)	187 (6.4%)	137 (9.7%)	0.69 (0.52;0.92)*	1.78 (1.35;2.33)***

*Note:* OR = odds ratio (adjusted for sex, age, educational attainment, living situation, and employment situation).

* *p* < 0.05.

** *p* < 0.01.

*** *p* < 0.001.

Odds ratios for low versus medium psychological well‐being additionally controlling for two or more SLEs were only slightly lower for any disorder (OR = 2.82, 95% CI: 2.35; 3.38), mood disorder (OR = 4.64, 95% CI: 3.71; 5.82), anxiety disorder (OR = 2.73, 95% CI: 2.23; 3.35), and substance use disorder (OR = 1.70, 95% CI: 1.29; 2.26), suggesting minimal overlap in explanatory value between ≥ 2 SLEs and poor well‐being.

### 3.3 Moderating Effect of Psychological Well‐Being

Table 4 shows the additive interaction effects of reporting at least two SLEs and psychological well‐being in relation to CMDs. For any CMD and all three separate types of CMDs, the proportion of people with a disorder was highest in those who experienced both ≥ 2 SLEs and low psychological well‐being. In this group, odds ratios ranged from 4.23 (95% CI: 2.97; 6.04) for substance use disorder to 27.01 (95% CI: 17.50; 41.71) for mood disorder. The pattern of odds ratios for this group was comparable for participants aged < 50 and ≥ 50 years, although values tended to be somewhat higher in the older group (Table 5).

**TABLE 4:** Moderating role of psychological well‐being in the association between stressful life events and common mood disorders in the total sample.

	Any disorder	Mood disorder	Anxiety disorder	Substance use disorder
	OR (95% CI)	OR (95% CI)	OR (95% CI)	OR (95% CI)
0–1 SLEs, high PW	Ref	Ref	Ref	Ref
0–1 SLEs, middle PW	1.34 (1.10; 1.64)	1.80 (1.27; 2.54)	1.21 (0.97; 1.52)	1.65 (1.23; 2.22)
0–1 SLEs, low PW	3.48 (2.75; 4.40)	8.55 (5.92; 12.33)	3.42 (2.74; 4.27)	2.75 (1.96; 3.87)
≥ 2 SLEs, high PW	1.80 (1.34; 2.41)	3.37 (1.68; 6.74)	1.51 (1.11; 2.05)	2.26 (1.43; 3.57)
≥ 2 SLEs, middle PW	2.82 (2.26; 3.52)	6.00 (3.88; 9.28)	2.56 (2.02; 3.25)	2.30 (1.65; 3.21)
≥ 2 SLEs, low PW	9.47 (7.04; 12.72)	27.01 (17.50; 41.71)	6.55 (4.68; 9.15)	4.23 (2.97; 6.04)

*Note:* OR = odds ratio (adjusted for sex, age, educational attainment, living situation, and employment situation).

Abbreviations: PW, psychological well‐being; SLEs, stressful life events.

**TABLE 5:** Moderating role of psychological well‐being in the association between stressful life events and common mood disorders stratified by age.

	Any disorder	Mood disorder	Anxiety disorder	Substance use disorder
	OR (95% CI)	OR (95% CI)	OR (95% CI)	OR (95% CI)
Aged < 50 years				
0–1 SLEs, high PW	Ref	Ref	Ref	Ref
0–1 SLEs, middle PW	1.23 (0.98; 1.54)	1.40 (0.86; 2.30)	1.06 (0.81; 1.39)	1.51 (1.09; 2.10)
0–1 SLEs, low PW	3.20 (2.34; 4.38)	7.26 (4.59; 11.49)	3.42 (2.56; 4.56)	2.10 (1.41; 3.14)
≥ 2 SLEs, high PW	1.72 (1.24; 2.39)	2.86 (1.21; 6.75)	1.47 (1.02; 2.12)	2.09 (1.28; 3.40)
≥ 2 SLEs, middle PW	2.59 (2.00; 3.35)	4.96 (3.03; 8.12)	2.26 (1.67; 3.06)	2.05 (1.40; 3.01)
≥ 2 SLEs, low PW	7.96 (5.34; 11.86)	24.54 (14.35; 41.96)	5.01 (3.41; 7.35)	3.20 (2.07; 4.94)
Aged ≥ 50 years				
0–1 SLEs, high PW	Ref	Ref	Ref	Ref
0–1 SLEs, middle PW	1.28 (0.84; 1.94)	3.04 (1.16; 7.95)	1.47 (0.96; 2.26)	1.73 (0.70; 4.24)
0–1 SLEs, low PW	3.14 (2.23; 4.42)	11.16 (3.83; 32.51)	3.45 (2.19; 5.46)	4.19 (1.82; 9.64)
≥ 2 SLEs, high PW	1.94 (1.15; 3.26)	6.38 (2.13; 19.12)	1.61 (0.78; 3.32)	4.10 (1.48; 11.36)
≥ 2 SLEs, middle PW	2.67 (1.79; 3.97)	9.10 (3.46; 23.97)	3.04 (1.97; 4.69)	2.94 (1.18; 7.30)
≥ 2 SLEs, low PW	8.70 (6.15; 12.30)	29.38 (10.93; 78.96)	8.39 (5.06; 13.93)	5.69 (2.33; 13.94)

*Note:* OR = odds ratio (adjusted for sex, educational attainment, living situation, and employment situation).

Abbreviations: PW, psychological well‐being; SLEs, stressful life events.

Additive interaction effects of SLEs and psychological well‐being were statistically significant for any CMD (RERI = 5.64, 95% CI: 3.18; 8.10, *p* < 0.001), mood disorder (RERI = 23.10, 95% CI: 10.10; 36.10, *p* < 0.001), and anxiety disorder (RERI = 3.45, 95% CI: 1.27; 5.64, *p* < 0.01), indicating a significant excess likelihood for these disorders for people with both ≥ 2 SLEs and low psychological well‐being. The additive interaction was not significant for substance use disorder (RERI = 0.21, 95% CI: −1.38; 1.80, *p* = 0.796). RERIs were similar for younger versus older age groups for any CMD (RERI = 5.13, 95% CI: 2.08; 8.18, *p* < 0.01 vs. RERI = 6.01, 95% CI: 3.04; 8.99, *p* < 0.001), but tended to be stronger for younger adults for mood disorder (RERI = 25.29, 95% CI: 8.85; 41.72, *p* < 0.01 vs. RERI = 14.68, 95% CI: 0.14; 29.23, *p* < 0.05) and stronger for older adults for anxiety disorder (RERI = 2.02, 95% CI: −0.23; 4.27, *p* = 0.078 vs. RERI = 5.31, 95% CI: 1.23; 9.39, *p* < 0.05). For both age groups, RERIs were non‐significant for substance use disorder (RERI = 0.43, 95% CI: −1.42; 2.28, *p* = 0.647 vs. RERI = −1.19, 95% CI: −5.07; 2.69, *p* = 0.548).

Additional sensitivity analyses in the total sample using the continuous Brief INSPIRE‐O scores, where higher scores indicate higher well‐being, indicated that only for the presence of any CMD was there a significant interactive effect with a negative RERI of −2.39 (95% CI: −4.42; −0.36, *p* = 0.021). RERIs were not significant for separate disorders of mood (RERI = −0.88, 95% CI: −2.52; 0.76, *p* = 0.293), anxiety (RERI = −0.80, 95% CI: −2.07; 0.47, *p* = 0.216) or substance use (RERI = 0.28, 95% CI: −0.47; 1.03, *p* = 0.460).

## 4 Discussion

The current study aimed to assess the relationship between stressful life events (SLEs) and the prevalence of common mental disorders (CMDs) in a large Dutch national representative sample. We were particularly interested in the potential moderating role of psychological well‐being as an integrated, overarching construct.

Our findings showed a significant relationship between experiencing at least two SLEs in the last year and a higher prevalence of all CMDs in the last year. Adults between 18 and 75 years of age who experienced at least two SLEs were almost 2.5 times more likely to suffer from any CMD in the past year, with the highest risk for mood disorder, with an adjusted odds ratio of 3.4. These findings are in line with earlier studies demonstrating an association between SLEs and the prevalence of CMDs (Clark et al. 2012; Jordanova et al. 2007; Newman and Bland 1994; Verplaetse et al. 2018). The highest risk for mood disorders may be explained by the fact that SLEs trigger sustained high levels of stress, which has been shown to be a robust risk factor for mood disorders (Hammen 2015).

The results also showed that the proportion of people with any CMD was the highest in adults who experienced both ≥ 2 SLEs and low psychological well‐being. This elevated likelihood of a CMD was similar for younger and middle‐aged to older adults, although RERIs indicated a somewhat stronger additive interaction effect for mood disorders and a weaker interaction effect for anxiety disorders for adults aged < 50 years. Although the current cross‐sectional study cannot establish any causal relationship, this finding suggests that people experiencing at least two SLEs may be especially vulnerable to developing a mood disorder when they also have low psychological well‐being. Strikingly, participants with ≥ 2 SLEs and low psychological well‐being were 27 times more likely to suffer from a mood disorder and over six times more likely to suffer from an anxiety disorder in the past year than those with < 2 SLEs and high psychological well‐being. For substance use disorder, having poor psychological well‐being didn't carry a significant excess risk.

These joint effect or interaction findings add to earlier studies that already demonstrated the predictive value of psychological well‐being regarding the prevention of the incidence of CMDs. For example, Wood and Joseph (Wood and Joseph 2010) found that people low in psychological well‐being were up to seven times more likely to meet the cutoff for clinical depression 10 years later. Schotanus‐Dijkstra and colleagues (2017) demonstrated that changes in well‐being were predictive of levels of psychopathology 3 and 6 months later, controlling for baseline levels of psychopathology. Using NEMESIS‐2 data from almost 4500 representative Dutch adults, Schotanus‐Dijkstra et al. (2017) found that the presence of high levels of mental well‐being at one moment reduced the risk of incident mood disorders by 28% and the risk of incident anxiety disorders by 53% three years later. Interestingly, similar to the current findings, associations with psychological well‐being appeared to be the weakest for substance use disorders.

One explanation for an additional vulnerability effect of low psychological well‐being on the relation between SLEs and mood and anxiety disorders is that diminished psychological well‐being reflects an absence of positive characteristics that promote successful coping with and adaptation to negative life events (Ryff 2013), such as positive and supportive relationships (Holeva et al. 2001), self‐acceptance (MacBeth and Gumley 2012), and purpose in life (Schnell and Krampe 2020). This also aligns well with recent models of mental health recovery that argue that complete recovery from mental health issues includes the presence of psychological well‐being (Bohlmeijer and Westerhof 2021).

Although several previous studies already demonstrated that specific psychological resources such as optimism, self‐acceptance, meaning in life, and mastery can buffer the effects of SLEs on specific psychopathological symptoms (Asselmann et al. 2017; Julian et al. 2021; Yu and Liu 2021; Wagner et al. 1996; Dulaney et al. 2018; Chou and Chi 2001), the current study is the first to examine the moderating role of psychological well‐being as a comprehensive construct on the relation between experiencing SLEs and meeting diagnostic criteria for a range of CMDs in a large and representative community sample. The current findings thus underscore the potential of promoting psychological well‐being as a preventive public (mental) health strategy in the general population. Over the past years, attention in mental healthcare and public health has gradually shifted toward promoting positive mental health alongside treatment or prevention of mental illness (Keyes 2007; Huppert 2009; Herrman and Jané‐Llopis 2012; Barry et al. 2019). Positive psychology interventions (PPIs), especially online self‐help PPIs, may allow for relatively easy, low‐cost, and widespread implementation and can potentially have a major impact on a population's well‐being (Bolier and Abello 2014). Although there is growing evidence that psychological well‐being can indeed be effectively improved with PPIs in both clinical and non‐clinical populations (Bolier et al. 2013; Weiss et al. 2016), their actual structural dissemination in the general population remains limited (Trudel‐Fitzgerald et al. 2019). Nonetheless, the current findings reinforce the notion that public health policies aimed at enhancing psychological well‐being may help foster more resilient populations and reduce the burden of mental illness.

### 4.1 Strengths and Limitations

A particular strength of this study is that it was conducted in a large representative national sample of the Dutch population (ten Have et al. 2023). Also, a structured diagnostic interview was used to determine DSM‐5 disorders (Kessler and Üstün 2004) while the Brief INSPIRE‐O, which was used to measure psychological well‐being, recently demonstrated construct validity, reliability, and measurement invariance across individuals with and without CMDs in the past year (Ruan et al. 2025). Together, these strengths enhance both the internal validity of the measurements and the generalizability of the findings.

An important limitation is the cross‐sectional nature of the current analyses, preventing any conclusions about temporal or causal mechanisms. Longitudinal analyses are warranted to further examine temporal associations and interactions between psychological well‐being, stressful life events, and common mental disorders over time. Moreover, because all three variables of interest were measured via self‐report, the study is subject to potential social desirability effects and other common method biases (Podsakoff et al. 2024). These biases may have led to under‐ or over‐reporting of SLEs, CMDs, and psychological well‐being, which in turn could have resulted in either underestimation or overestimation of the true associations among these variables. Also, all analyses concerning the associations between SLEs, CMDs, and psychological well‐being were adjusted for sociodemographic factors, but we did not consider potentially relevant contextual factors like history of mental disorders or treatment. Especially, previous mental disorders may play a significant role in the relationship between SLEs, psychological well‐being, and CMDs. Future studies could specifically address this in a longitudinal setting by focusing on new‐onset disorders versus recurrent or persistent disorders. Finally, arbitrary data‐driven tertiles of the Brief INSPIRE‐O scores were used to classify participants as having low, medium and high levels of psychological well‐being as no established cutoffs are available for defining poor or good psychological well‐being on this instrument. The finding that the RERIs for the different CMDs in the sensitivity interaction analyses with the continuous Brief INSPIRE‐O scores were not significant suggests that the interaction effect may not be strong or consistent across the entire range of psychological well‐being, but only at particular (in this case, very low) levels of well‐being. Future research could further explore this to identify specific thresholds for poor psychological well‐being, as measured by the Brief INSPIRE‐O, that can optimally distinguish individuals at heightened risk for mental disorders when faced with stressful life events.

## 5 Conclusion

The current study confirmed that experiencing multiple SLEs in the past year is associated with CMDs. The findings add to the literature in showing that the combination of ≥ 2 SLEs and poor psychological well‐being is associated with a substantially higher prevalence of anxiety disorder and especially mood disorder. This suggests that promoting psychological well‐being may be a fruitful public mental health strategy to increase resilience against SLEs.

## Author Contributions

**Peter M. ten Klooster:** conceptualization, methodology, writing – original draft preparation, writing – review and editing. **Margreet ten Have:** conceptualization, data curation, methodology, formal analysis, writing – review and editing. **Annemarie I. Luik:** data curation, methodology, writing – review and editing. **Marlous Tuithof:** data curation, formal analysis, writing – review and editing. **Ernst T. Bohlmeijer:** conceptualization, methodology, writing – original draft preparation, writing – review and editing.

## Funding

NEMESIS‐3 is conducted by the Netherlands Institute of Mental Health and Addiction (Trimbos Institute) in Utrecht. Financial support has been received from the Dutch Ministry of Health, Welfare, and Sport. The funding source had no further role in study design; in the collection, analysis, and interpretation of data; in the writing of the current report; or in the decision to submit the paper for publication.

## Ethics Statement

The Medical Research Ethics Committee Utrecht determined that NEMESIS‐3 did not fall within the remit of the Dutch Medical Research Involving Human Subjects Act (reference number: WAG/mb/19/017126; May 15, 2019). Therefore, no formal medical‐ethical approval was required. The field procedures, information for respondents, and informed consent forms were assessed positively by the local ethical review committee.

## Conflicts of Interest

The authors declare no conflicts of interest.

## Data Availability

The data on which this manuscript is based are not publicly available. However, data from NEMESIS are available upon request. The Dutch Ministry of Health financed the data and the agreement is that these data can be used freely under certain restrictions and always under the supervision of the Principal Investigator (PI) of the study. Thus, some access restrictions do apply to the data. The PI of the study is the second author of this paper and can at all times be contacted to request data. Researchers can contact the PI of NEMESIS and submit a research plan, describing its background, research questions, variables to be used in the analyses, and an outline of the analyses. If a request for data sharing is approved, a written agreement will be signed stating that the data will only be used for addressing the agreed research questions described and not for other purposes.
